# Towards Higher Sensitivity of Mass Spectrometry: A Perspective From the Mass Analyzers

**DOI:** 10.3389/fchem.2021.813359

**Published:** 2021-12-21

**Authors:** Chang Li, Shiying Chu, Siyuan Tan, Xinchi Yin, You Jiang, Xinhua Dai, Xiaoyun Gong, Xiang Fang, Di Tian

**Affiliations:** ^1^ College of Instrumentation & Electrical Engineering, Jilin University, Changchun, China; ^2^ Technology Innovation Center of Mass Spectrometry for State Market Regulation, Center for Advanced Measurement Science, National Institute of Metrology, Beijing, People’s Republic of China

**Keywords:** mass spectrometry, mass analyzers, sensitivity, quadrupole, ion trap, time-of-flight, Fourier transform ion cyclotron

## Abstract

Mass spectrometry (MS) is one of the most widely used analytical techniques in many fields. Recent developments in chemical and biological researches have drawn much attention to the measurement of substances with low abundances in samples. Continuous efforts have been made consequently to further improve the sensitivity of MS. Modifications on the mass analyzers of mass spectrometers offer a direct, universal and practical way to obtain higher sensitivity. This review provides a comprehensive overview of the latest developments in mass analyzers for the improvement of mass spectrometers’ sensitivity, including quadrupole, ion trap, time-of-flight (TOF) and Fourier transform ion cyclotron (FT-ICR), as well as different combinations of these mass analyzers. The advantages and limitations of different mass analyzers and their combinations are compared and discussed. This review provides guidance to the selection of suitable mass spectrometers in chemical and biological analytical applications. It is also beneficial to the development of novel mass spectrometers.

## Introduction

Recent developments in chemical and biological researches have drawn much attention to the measurement of low-abundance substances in samples, such as biomarkers and single-cell metabolites. These low-abundance substances often play important roles in their corresponding chemical or biological systems (Chen et al., 2016; Finn et al., 2020; Marabelle et al., 2020). Accurate and sensitive analysis of these low-abundance substances is essential for the in-depth interpretation of related chemical and biological phenomena (Sonnett et al., 2018; Yin et al., 2018; Liang et al., 2019). Therefore, there are great demands for analytical instruments with higher sensitivity. Mass spectrometry (MS) has unparalleled sensitivity and specificity. It is one of the most widely used analytical techniques in many fields, such as analytical chemistry (Donnelly et al., 2019), life sciences (Zenobi, 2013; Lehmann, 2017), environmental monitoring (Hollender et al., 2019) and food safety (Shao et al., 2019). Continuous efforts have been made consequently to further improve the sensitivity of MS so as to meet the analytical requirements in different fields.

Sensitivity is an index reflecting the performance of mass spectrometers. The sensitivity of mass spectrometers can be expressed in the way of signal-to-noise ratio (S/N) (Ingle, 1974). S/N is defined as the ratio of signal intensity (S) to noise (N) obtained for a certain amount of sample (Gross, 2011). Thus, it can be improved by either increasing the signal intensity or reducing the noise. The higher S/N represents the higher sensitivity for a certain amount of sample. In addition to S/N, limit of detection (LOD) and limit of quantification (LOQ) are also common expressions of sensitivity. LOD is defined as the lowest concentration of the component to be reliably detected in the sample, and the S/N is 3:1 (Nijat et al., 2020). LOQ is the lowest concentration of the component to be reliably quantified in the sample, and typical S/N ratio is 10:1. The LOD and LOQ can also represent the detection capability of mass spectrometers. The lower LOD and LOQ reflect the higher sensitivity of mass spectrometers.

The improvement of mass spectrometers’ sensitivity can be achieved in different ways. The optimization of the pretreatment procedures is the most commonly used way. It is difficult to directly measure low-abundance substances in complex matrices (Torres et al., 1996; Ahmed, 2001). The interferences of the matrices can be reduced effectively by the use of pretreatment techniques. The pre-processed samples can easily be determined by subsequent MS analysis, thereby reducing the LOD of the analytical methods (Jakubowska et al., 2005; Cruickshank-Quinn et al., 2014). The development of novel ionization techniques is an extremely effective way. Continuous efforts have been paid in recent years on the development of novel ionization sources, especially ambient ionization sources. Techniques have been developed to increase the ionization efficiency of targeted substances, thereby increasing the signal intensities and sensitivity. In-source techniques have also been developed to remove complex matrices in biological samples. The interferences of matrices are significantly eliminated and the S/N of analytes is improved. Developments in the ion transmission systems improve the transmission efficiency of ions from the ambient atmosphere to the mass analyzers. Novel ion funnel and ion lens were developed to focus and transfer ions more efficiently (Zhai et al., 2019; Snyder et al., 2020). The transmission efficiency of ions can be improved with these techniques. The ion beam can be radially confined more tightly by using a quadrupole ion guide (Chen et al., 2015). The radial expansion of the ion cloud is reduced. The transmission efficiency and mass selection accuracy of ions are improved. Complex mixtures can be measured more sensitively.

The improvement of mass analyzers is a promising way. In summary, there are four basic strategies used on mass analyzers for the improvement of mass spectrometers’ sensitivity, including the improvement of ion transmission efficiency, the selective enrichment of targeted ions, the improvement of the ion utilization rate and the improvement of the signal-to-noise ratio (S/N) of the spectrum. The strategy of improving the ion transmission efficiency is mainly used in quadrupole mass analyzers. The strategy of selectively enriching targeted ions is mainly used in ion trap mass analyzers. The strategy of improving the ion utilization rate is used in time-of-flight (TOF), Fourier transform ion cyclotron (FT-ICR) and Orbitrap mass analyzers. The strategy of improving the S/N of the spectrum is mainly used in quadrupole tandem mass analyzers.

Till now, there have been many reviews that summarize the improvement of pretreatment procedures and ionization techniques (Raterink et al., 2014; Klampfl and Himmelsbach, 2015; Peacock et al., 2017; Deng et al., 2019). Few reviews have focused on the improvement of mass analyzers. However, mass analyzer is the core component of a mass spectrometer. The betterment of mass analyzers is the most direct, universal and practical way to enhance the sensitivity of MS. At present, excellent works have been down on the improvement of different types of mass analyzers to increase the sensitivity of MS. But few papers provide a complete and systematic review of these works. This paper reviews the recent developments of mass analyzer techniques that improve the sensitivity of MS. It summarizes the development trends of different mass analyzers and their combinations for higher sensitivity, including quadrupole, ion trap, time-of-flight (TOF) and Fourier transform ion cyclotron (FT-ICR) analyzers, along with different combinations of these analyzers. The review offers us new insights into the development trends of mass spectrometers from a perspective of mass analyzers. It provides guidance to the selection of suitable mass spectrometers in chemical and biological analytical applications. It is also beneficial to the development of novel mass spectrometers.

## Quadrupole Mass Analyzer

Quadrupole mass analyzer, also known as quadrupole mass filter (QMF), is composed of four parallel cylindrical or hyperbolic rods. Theoretically, an ideal hyperbolic electric field can be obtained by ideal hyperbolic electrodes. In practice, the processing and assembly of ideal hyperbolic rods are of great difficulty. Thus, cylindrical electrodes are often used instead of hyperbolic electrodes in quadrupole.

There are two working modes for the quadrupole mass analyzer, namely the ion guide mode for ion transfer and the mass filter mode for mass analysis. The mechanisms behind the two modes are different. In the ion guide mode, the quadrupole is operated in a radio frequency (RF)-only mode without direct current (DC) voltage, ions with a wide range of mass-to-charge ratio (m/z) values have stable trajectories in the quadrupole (Douglas, 2009). Under this condition, the quadrupole acts as an ion guide, transmitting ions generated from the ambient ionization source to the mass analyzer in high vacuum. There is a vacuum gradient from the outer side of the quadrupole to the inner side, ranging from 0.1 to 1 × 10^–3^ mbar.

In the mass filter mode, a DC voltage is applied to one opposite pair of electrodes of the quadrupole while a RF voltage is applied to the other opposite pair of electrodes. The voltage polarity between two adjacent electrodes is opposite (Miller and Denton, 1986). The DC and RF voltages generate a quadrupole field within the quadrupole. The movement of ions in the quadrupole field can be described by the first stable zone diagram. Ions with different m/z can be scanned/filtered by changing the DC and RF voltages (March 1997). In this review, we mainly focus on the mass filter mode. This is how the quadrupole acts as a mass analyzer.

Quadrupole has the advantages of simple structure and low cost. It can be used in fields such as atmospheric monitoring and aerospace (Franz et al., 2014; Franz et al., 2017; Trainer et al., 2019). The “Curiosity” landing on Mars is equipped with a quadrupole mass analyzer that can measure light isotopes and volatiles (Mahaffy et al., 2012). The measurement results confirmed the presence of chlorobenzene and similar organics on Mars. Due to the limitations in mass range and resolution, it is not conducive for quadrupole to analyze macromolecular compounds. In addition, qualitative analysis is another drawback of quadrupole, due to its inability to perform MS^n^. The combination of quadrupole with other mass analyzers offers an alternative way to make up for its drawbacks. Quadrupole is often placed in front of other mass analyzers to filter ions for the purpose of removing matrix ions.

Introducing a delayed DC ramp to quadrupole can improve the transmission efficiency of the quadrupole (Brubaker, 1968). A short RF-only quadrupole (pre-filter) is placed in front of the main quadrupole with RF and DC supply. The DC voltage is applied to the main quadrupole only after the ions enter the main quadrupole. Thus, the a and q parameters of the targeted ions always remain in the stable region. Losses of ions caused by electric fringe fields at the entrance of the main quadrupole are reduced. The sensitivity is significantly improved. This method is used in many modern commercial mass spectrometers. However, it is not useful when using higher stability regions. Huntley and Reilly (2020) simulated the acceptance and transmittance of mass filters driven by sine and rectangular waveforms. When a pre-filter was used or the duty cycle was delayed, the sensitivity of both systems is increased by four times. The ions directly reached the apex of the stability region when the pre-filter was not used. The ions were unstable along the *y*-axis, leading to ion loss. Putting a pre-filter in front of the main quadrupole can physically achieve a delayed DC field. Initially, the ion only experienced a monotonic increase to the value of q due to the absence of DC, i.e., a = 0. The q value of the ion was increased to a value under the apex. Subsequently, the a value of the ion was increased rapidly until the ion reached the vicinity of the apex. This ensured that the path of the ion to the apex of the stability region was stable on both axes. Thus, it improved the sensitivity. The rectangular waveform based on the frequency dynamic duty cycle drove the mass filter without applying a DC voltage. Adjusting the duty cycle could narrow the range of stable q values. The mass distinction was achieved by moving from the apex to a = 0 axis. Ions moved along a stable path. The transmission efficiency of the quadrupole was improved.

However, the addition of the pre-filter also causes the reflection of ions at the boundary of the pre-filter/quadrupole. Ions are trapped in the pre-filter region (Dawson, 1975). Loss of ions in the mass filter is caused. Collings (2019) modified the pre-filter. The field radius of the pre-filter rods was reduced to match the effective potentials at the boundary of the pre-filter or mass filter. The effective potential of the pre-filter was greater than that of the mass filter to generate an effective potential gradient at the boundary. In addition, the pre-filter was rotated about its longitudinal axis. The reflected ions entered an unstable ion trajectory. Finally, the pre-filter rods were tilted to form the effective potential gradient toward the length of the pre-filter rods. These improvements have greatly reduced the number of ions trapped in the pre-filter.

Some works make use of stable islands to increase the ion transmission efficiency. An auxiliary quadrupole field is applied to main quadrupole. Resonance excitation induced ion oscillations caused many bands of instability on both sides of the first stability region, forming many stable islands (Konenkov et al., 2001). Each stable island has its own operation point and can be used for ion separation. Zhao et al. (2010) increased the transmission efficiency by about two orders of magnitude by using stable islands on a conventional cylindrical quadrupole. Two additional alternating current (AC) excitations were used by Sudakov et al. (2016) to form a narrow and long stability band along the X-band at the right of first stability region. The X-band is similar to the higher stability region. The delayed DC ramp technique can be combined to enhance the sensitivity. Douglas and Konenkov (2018) developed a new mode of the main quadrupole operation. A constant dipole voltage was applied between the y electrodes of main quadrupole. An auxiliary RF quadrupole voltage was applied to the x and y electrodes. The modification of the stability diagram was studied. The low-mass tail of a peak could be removed to control the resolution, so that the isotopic abundance sensitivity was improved by about two orders of magnitude.

Xue et al. (2021) combined a soft ambient ionization technique with enhanced in-source fragmentation and multiple fragment ion monitoring technique on a single quadrupole mass spectrometer for high-sensitivity quantitative analysis. It can be used to determine standard mixtures and metabolites in cell and plasma extracts. The single quadrupole mass spectrometer has comparable quantitative performance with triple quadrupole (QqQ) mass spectrometer, and can even detect certain chemical substances more effectively. Compared with multiple reaction monitoring (MRM) of the QqQ, single quadrupole multiple fragment ion monitoring analysis of C20 sphingosine is more sensitive. The dynamic range is extended to lower analyte concentrations.

## Ion Trap Mass Analyzer

Wolfgang Paul and his coworkers first proposed a three-dimensional (3D) ion trap mass analyzer, which had two hyperboloid end-cap electrodes and a ring electrode (Paul and Steinwedel, 1953). The ion trap can focus the ions at the center and realize the selection and detection of specific m/z ions in the trap (Snyder et al., 2016a). The ion trap mass analyzer has become the first choice for the development of miniaturized mass spectrometers due to its simple structure, small size, high sensitivity and relatively lower vacuum requirements. It can isolate ions and realize MS^n^. It is suitable for the qualitative research of molecular structure (Frankfater et al., 2019). The ion trap mass analyzer has played an important role in expanding the field of proteomics (Mayya et al., 2005; Blackler et al., 2006). It is also used to investigate the chemical reaction of gas phase ions (Guo et al., 2015; Parchomyk and Koszinowski, 2019). But there are strong Coulomb interactions among the large number of ions trapped in the ion trap, resulting in space charge effects. The accumulation of ions is limited (Hager, 2002).

A two-dimensional (2D) linear ion trap (LIT) has been developed (Hager, 2002; Schwartz et al., 2002). Compared with 3D ion trap, ions are not axially restricted by the RF potentials in the 2D ion trap. The trapping efficiency of LIT is about 10 times that of quadrupole ion trap (QIT), and the charge capacity is 20 times that of QIT (Douglas et al., 2005; Riter et al., 2006). In order to facilitate the manufacture and miniaturization, 3D ion traps with simplified geometric shapes were developed. The reduction in trapping capacity and sensitivity caused by the decreased physical size of the ion trap is compensated. The cylindrical ion trap (CIT) consists of a cylindrical ring electrode and two flat end-cap electrodes (Patterson et al., 2002). It is much easier to fabricate than the conventional ion trap with hyperbolic shaped electrodes. Toroidal RF ion trap is another choice. It has higher ion storage capacity and is easy to miniaturize (Lammert et al., 2001). The storage capacity is increased by trapping ions in a ring.

Cooks et al. developed a rectangular ion trap (RIT) (Ouyang et al., 2004). Rectangular geometry electrode pairs were used to build a trap to increase the ion trapping capacity (Ouyang, et al., 2004; Peng et al., 2008; Song et al., 2006). Due to the higher ion capture capability of RIT, the S/N was increased by 40 times compared to the conventional cylindrical ion trap. Huo et al. (2020) modified a conventional RIT electrode into an asymmetric rectilinear ion trap (ARIT) with unidirectional ion ejection capability. Convex and concave circular structures were added to the two X electrodes. The electric field center of the ion trap was inclined to the concave side. The trapped ions were collected near the concave side where an ion detector was installed. It can detect a stronger ion signal than the conventional RIT. The detection limit for rotundine reached 1-ppb level. There is no obvious decrease of the dynamic range and mass resolution.

Digital waveform technology (DWT) is introduced to ion trap to instantaneously switch the frequency and duty cycle of the waveform. In digital ion trap (DIT) (Ding et al., 2002; Ding and Brancia, 2006; Brancia et al., 2010), a rectangular waveform is used to drive the ion trap. Mass selective ion ejection and excitation can be performed by scanning the frequency of the rectangular waveform. DIT has many advantages, such as low trapping voltage and high-quality analysis capabilities. It also has lower power requirements than the ion traps using sine waveforms. More efficient fragmentation can be achieved, which provides higher sensitivity and more molecular structure information.

**FIGURE 1:**
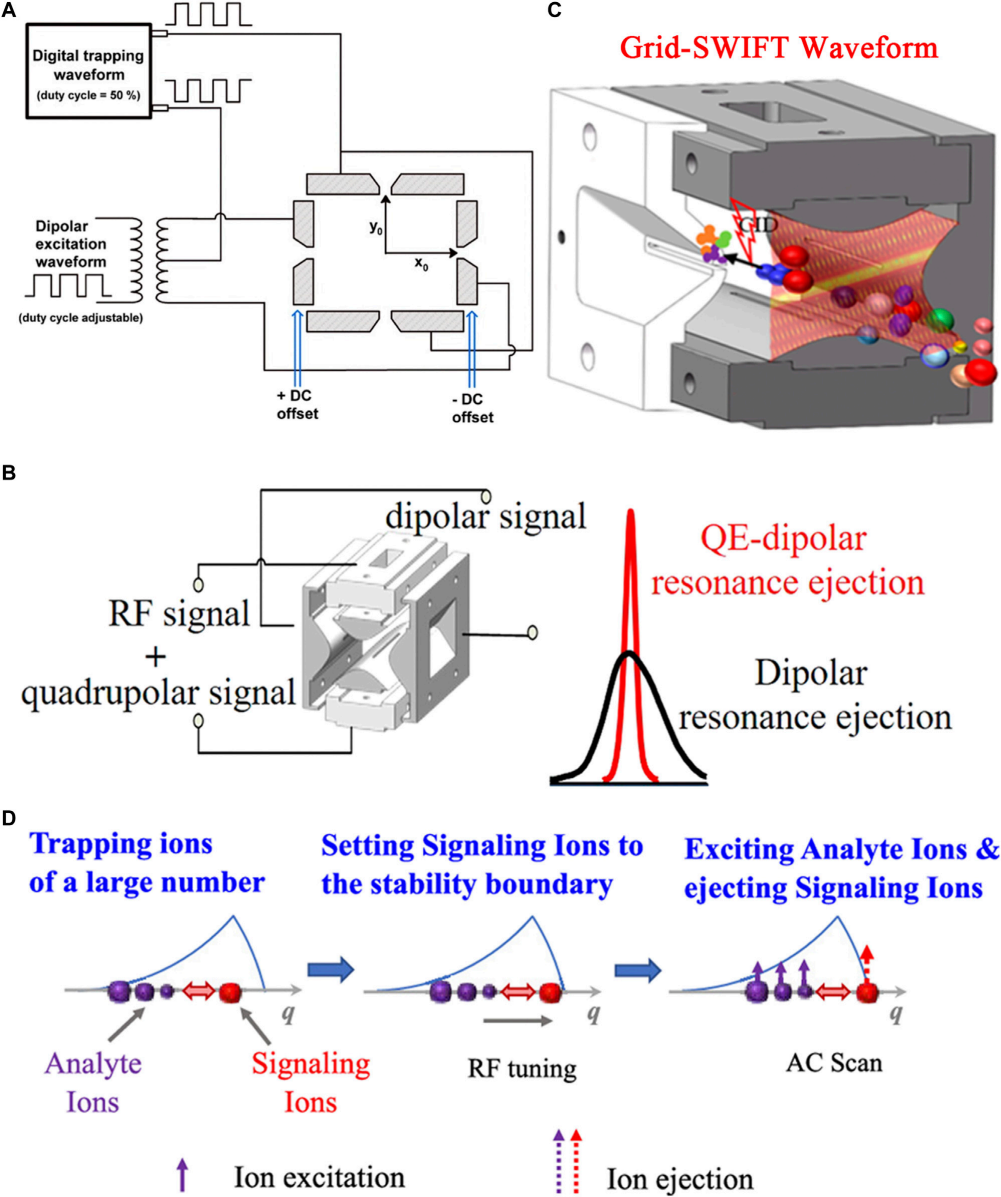
Waveform applied on the ion trap. **(A)** Schematic of circuit for a ceramic-based linear ion trap (cRIT). Adapted with permission from Wang, L., Xu, F. and Ding, C.-F (2013). Anal. Chem. 85, 1,271–1,275 (Wang, et al., 2013). Copyright 2013 American Chemical Society; **(B)** Schematic diagram of LIT applied signal. Adapted with permission from Jiang, T., Xu, Q., Zhang, H. J., Li, D. Y. and Xu, W (2018). Anal. Chem. 90, 11,671–11,679 (Jiang et al., 2018). Copyright 2018 American Chemical Society; **(C)** The Grid-stored waveform inverse Fourier transform (Grid-SWIFT) waveform in the “brick” MS. Adapted with permission from Xu, Z. Q., Jiang, T., Xu, Q., Zhai, Y. B., Li, D. Y. and Xu, W. (2019). Anal. Chem. 91, 13,838–13,846 (Xu et al., 2019). Copyright 2019 American Chemical Society; **(D)** Schematic illustration for mass analysis using collective interaction. Adapted with permission from Zhou, X. and Ouyang, Z (2021). Anal. Chem. 93, 5,998–6,002 (Zhou and Ouyang, 2021). Copyright 2021 American Chemical Society.

Wang et al. (2013) used rectangular waveforms for ion trapping in a ceramic-based linear ion trap (cRIT) system. A dipolar excitation waveform was formed by dividing down the trapping rectangular waveform (Figure 1A). It is applied to a pair of x electrodes for ion ejection. Highly efficient CID are performed by changing the duty cycle of the excitation waveform. Xu et al. (2014) further developed a novel ion activation method by using a rectangular wave dipolar potential. The frequency of the rectangular wave dipolar potential formed by dividing down the trapping rectangular waveform is adjusted. The selected precursor ions are resonance excited to high kinetic energy and then dissociated. Xu et al. (2016) continued to study the effects of experimental parameters on the efficiency of CID such as trapping waveform frequency and amplitude. The efficiency of CID is higher when the voltage of the trapping waveform is higher. Xue et al. (2016) developed a portable digital linear ion trap (LIT) mass spectrometer for the analysis of volatile organic compounds (VOCs). A digital waveform was used to capture and excite ions. It is especially suitable for on-site monitoring. The corresponding LOD of the mixed gas samples of benzene, toluene, xylene and monochlorobenzene measured are 0.67, 0.32, 0.19 and 0.37 ppbv, respectively.

Two DC potentials with the same amplitude but different signs can be applied on the pair of x-electrodes with ejection slits to form a dipolar DC field (Tolmachev et al., 2004). He et al. (2017) developed a dual-polarity LIT mass spectrometer. An additional dipole DC field was applied to the ejection electrodes of the LIT. Cations and anions could be simultaneously operated and analyzed. The DC was adjusted to control the spatial distribution of cations and anions. The ions in the center of the LIT are moved to the RF electrode, achieving the directional ion ejection. Compared with the conventional ion ejection mode, the signal intensity of ions was improved by 2 times, and the ejection and detection efficiency of ions could also be enhanced by about one-fold. More structural information can be obtained by simultaneously obtaining the fragment ions of the cation and anion of the peptide. Ions can be distinguished by their charge polarity. Ion/ion reaction kinetics process can be controlled and monitored in real time.

By applying a DC voltage on the ejection electrodes in a LIT MS, the ions are moved from the central axis of the four electrodes to near the eject slit and were more easily ejected (Li et al., 2018). Xu’s group proposed a quadrupole enhanced (QE) dipolar resonance ejection method which was implemented in their home-built “brick” mass spectrometer (Jiang et al., 2017; Jiang, et al., 2018). A quadrupolar excitation signal was coupled with a RF signal to form a combined signal (Figure 1B). The combined signal was applied on one pair of the hyperbolic electrodes of the LIT, while a dipolar excitation signal was applied on the other pair of the hyperbolic electrodes. The ion ejection point could be controlled by adjusting quadrupole and dipole excitation frequencies. Compared with dipole resonance ejection, QE dipolar resonance ejection can increase the ion intensity, thereby improving the detection sensitivity by no less than 2 times. The LOQ was 0.25 μg/ml using the QE-dipolar resonance ejection method. A mixture of the peptide Met-Arg-Phe-Ala (MRFA, 100 μg/ml) and reserpine (Res, 50 μg/ml) and bradykinin (100 μg/ml) were analyzed using a Brick mass spectrometer. The resolution and signal intensity are enhanced. In addition, the faster ion ejection during the QE-dipole resonance ejection process could shorten the duration time of the Coulomb interaction during the ion ejection process. The space charge effect in the ion trap was reduced. This method can be widely applied to benchtop and miniaturized ion trap mass spectrometers. A triple resonance method was developed to perform precursor ion scan to improve sensitivity (Szalwinski et al., 2020). The excitation of the precursor ions and the first-generation (MS^2^) product ions and the ejection of the second-generation (MS^3^) product ions occur simultaneously. Species that produce product ions with the same m/z but different structures are identified by MS^3^ product ions. When using the triple resonance method for precursor ion scan of chemical warfare agent simulants (cyclohexyl methylphosphonate and pinacolyl methylphosphonate) and central nervous system stimulants (amphetamine and methamphetamine), the increases in sensitivity can be observed.

Space charge effects can significantly limit the sensitivity of all the ion trap mass spectrometers. Techniques are developed to reduce the impact of space charge effects. Dalton et al. used a dual-frequency waveform that contained two closely spaced frequencies (Snyder and Cooks, 2016b). Their relative amplitudes were adjusted. The first frequency excited the ions to higher amplitudes where space charge effects were less prominent. When the ions come into resonance with the second frequency, the ions were more effectively ejected from the ion trap. Compared with conventional single frequency resonance ejection, the resolution and sensitivity are improved. Thus, successive resonances may be most applicable to improving the performance of miniature mass spectrometers. The ions trapped in the ion trap can be effectively isolated by applying a stored waveform inverse Fourier transform (SWIFT) on the electrodes of the ion trap. The space charge effect is reduced to a certain extent (Chen et al., 1987; Guan and McIver, 1990; Guan and Marshall, 1996). However, the target ions cannot be isolated during the ion injection period. Xu’s group constructed a broadband ion excitation signal, called a Grid-SWIFT waveform (Xu, et al., 2019), on the “brick” MS. The Grid-SWIFT waveform had a grid shape time-frequency domain spectrum and multiple frequency components present at any time (except in the notch area). Only the ions of interest were isolated into the ion trap by continuously ejecting unwanted ions during ion injection period (Figure 1C). Interferences of solvents and impurities were removed. Space charge effects were reduced. The selectivity and S/N of the target analytes were improved. Compared with the conventional SWIFT waveform, the detection sensitivity of the targeted ions could be improved by about 4 times. The operation process similar to that of a QqQ was realized on the “brick” MS. The “brick” MS can be used for the quantification of target analytes. In the selected ion monitoring (SIM) mode, a LOD of 50 ng/ml is obtained by using Grid-SWIFT. In the pseudo-selected reaction monitoring (Pseudo-SRM) mode, a LOD of 50 ng/ml and RSD less than 12% was obtained over a concentration range of 100–1,000 ng/ml. In the pseudo-multiple ion monitoring (pseudo-MIM) mode, a good linearity is obtained (correlation coefficient of *R* ^2^ > 0.99), and an RSD is better than 11%. The performance achieved on the “brick” mass spectrometer makes on-site quantitative analysis possible.

Mass analyzers manipulate the ions based on gas hydrodynamic (H) field and electric (E) field (Zhou and Ouyang, 2016). The effect of H field and space charge of E field are important for the design of mass analyzers. Zhou and Ouyang (2014) developed an electro-hydrodynamic simulation (EHS) method that combines the E field and H field to simulate ion trajectories. A dynamic gas field can be generated in the instrument when ions are transferred between two regions with different pressures. The transfer of externally introduced ions to the LIT was studied by using the EHS method with the dynamic gas field. It was also verified by experimental characterization. Due to the gas expansion, 100% of the ions had at least one collision and more than 65% of them had multiple collisions. Therefore, the low-pressure LIT has a relatively high trapping efficiency. Studies have shown that the gas expansion in high vacuum has a great influence on ion transfer and mass analysis, thereby affecting the sensitivity. The EHS method has great potential in the design of hybrid instruments and the research of ion energetics.

Zhou and Ouyang (2021) used a collective interactions of the ions for mass analysis (Figure 1D). Analyte ions and signaling ions were first stored in the trap. Then, the signaling ions were placed close to the stable boundary by adjusting the RF. Compared with the signaling ions, the analyte ions were in a deeper potential well. Finally, the analyte ions were excited by the alternating current (AC). The excitation energy of the analyte ions was transferred to the signaling ions through collective interaction. The signaling ions were ejected from the LIT. The EHS method was used to simulate ion trajectories. The collective interaction process was further discussed. Mass analysis using collective interaction of ions is nondestructive to the analyte ions. The collective interaction ejection can increase the signal intensity of the analyte ions. It can be used for identification of charge states of proteins.

**FIGURE 2:**
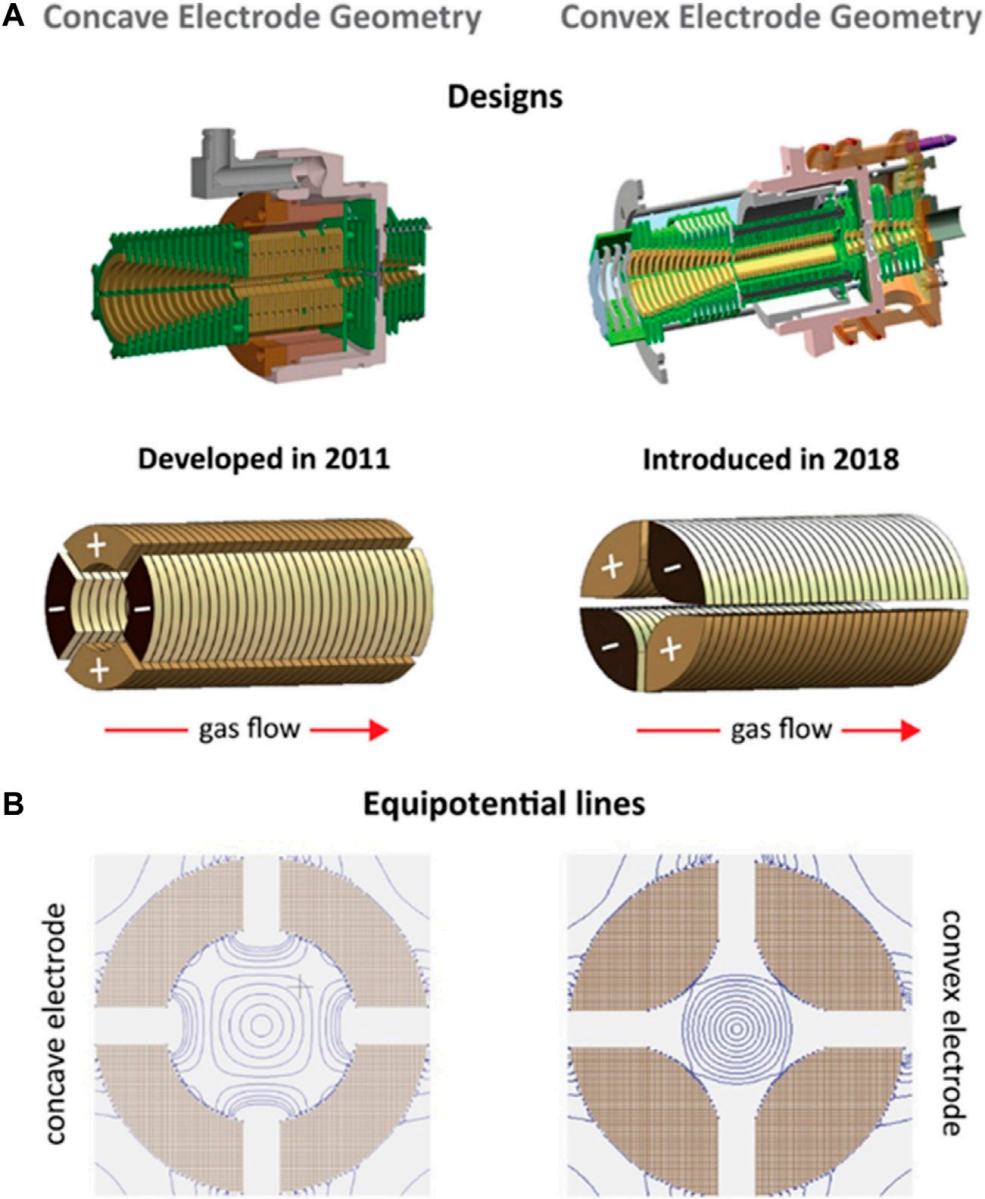
Designs and simulations of TIMS. **(A)** Designs of the concave and convex electrode geometries; **(B)** Equipotential lines of the concave and convex electrode geometries at a given applied voltage. Adapted with permission from Jeanne Dit Fouque, K., Garabedian, A., Leng, F., Tse-Dinh, Y.-C., Ridgeway, M. E., Park, M. A., et al. (2021). Anal. Chem. 93, 2,933–2,941 (Fouque et al., 2021). Copyright 2021 American Chemical Society.

Trapped ion mobility spectrometry (TIMS) utilizes the synergy of E field force and gas flow to achieve ion mobility separation. The complexity of the spectrum is reduced. The sensitivity is increased. The TIMS device is composed of three parts, including an entrance funnel, the TIMS tunnel and an exit funnel (Fernandez-Lima F. et al., 2011; Fernandez-Lima F. A. et al., 2011). Ions are focused into the TIMS tunnel and move forward under the push of the gas flow. Ions are separated according to their cross sections. The direction of the E field applied on the TIMS funnel is opposite to the direction of the ion movement, preventing the forward movement of ions. The ions are trapped in the specific location. Then the ions are released by gradually reducing the E field strength (Michelmann et al., 2014; Silveira et al., 2016). Fouque et al. (2021) developed a novel TIMS for the analysis of macromolecular assemblies. The TIMS analyzer used a convex electrode (Figure 2). The pseudopotential penetration in the radial dimension was increased. The mobility trapping was extended to high-molecular-weight species in native state. It is widely used in the analysis of intrinsically disordered proteins and functional proteomics.

## TOF Mass Analyzer

The ions formed in the ion source obtain the initial kinetic energy from the accelerating voltage. The ions then enter the field-free drift spatial region. Ions with different m/z have different flight time. The ions are separated according to their m/z. Ions with the smaller m/z arrive at the end of the drift length before ions with a larger m/z (Wiley and Malaren, 1955). The TOF mass analyzer has the advantages of fast analysis speed and high upper limit of quality (Bolaños et al., 2003; Vestal, 2009). It is especially suitable for the analysis of large-throughput and fast analysis of biological macromolecules (Karas, 1990; Park et al., 2012; Ramandi et al., 2021). The TOF mass analyzer can measure the mass of many peptides simultaneously. It has become the most popular tool for peptide mass fingerprint analysis (Alnakip et al., 2020; Sun et al., 2020). However, a relatively high degree of vacuum and a relatively constant temperature environment are required. MS^n^ can be achieved by TOF through the combination of collision devices in front of the TOF mass analyzer (Cotter et al., 2004).

The reflectron is an effective means for TOF focusing of ion packets through energy. The use of gridless ion reflector can avoid the scattering of ions on the grids of the ion reflector. Multi-reflection time-of-flight (MR-TOF) mass analyzer with multiple gridless mirrors was developed (Plaß et al., 2013). The total length of the mass spectrometer is reduced by folding the ion path between multiple gridless mirrors. Further improvements in the ion optics lead to the increase of the energy and spatial acceptance of the MR-TOF mass analyzer, improving the mass resolution and sensitivity. Yavor et al. (2008) described a MR-TOF analyzer with a jig-saw ion path. Ions move along the jig-saw path between two parallel planar gridless ion mirrors. The design of high-quality four-electrode planar gridless ion mirrors realize the 3rd-order TOF focusing with respect to ion energy. The 2nd-order TOF focusing with respect to spatial spread in the direction normal to the plane of the jig-saw motion is also achieved. This design of the mass analyzer allows the analysis of ions in the full mass range. Yavor et al. (2015) developed an optimized high-performance axially symmetric four-electrode gridless ion mirrors. The developed MR-TOF MS was successfully applied to the study of exotic nuclides. Spivak-Lavrov et al. (2020) designed multi-stage and multi-reflective TOF analyzers with wedge-shaped mirrors. An inhomogeneous accelerating field was generated by changing the potentials on the electrodes of the mirror. Packets of ions with identical masses were compressed in the direction of their movements. The detector was placed in the largest plane where the ion packet was compressed. The high resolution and sensitivity of TOF MS can be achieved.

Multi-turn (MT) TOF offers an alternative way to extend the flight pass of ions. Shchepunov et al. (2019) proposed a design of the ion optics for a high-resolution MT-TOF mass analyzer. The mass analyzer has rotationally symmetric main electrodes and O-type closed planar orbits. In order to improve transmission efficiency, the electric field of the mass analyzer is configured to achieve focusing in two lateral directions. Yavor et al. (2019) proposed a design of the ion optics with a sector field for MT-TOF mass analyzer. The mass analyzer has cylindrical electrostatic sectors and a periodic array of two-dimensional lenses. Oval-type spiral ion trajectories can be formed. It has a 2nd-order TOF focusing with respect to the energy and space (coordinates and angles) in the ion packet.

In modern TOF mass spectrometers, most of the ions are lost in the extraction area, and the duty cycle is usually between 5 and 30% (Loboda and Chernushevich, 2009). Insufficient utilization of ions limits the sensitivity of the mass spectrometers. The ion throughput and sensitivity can be improved by increasing the duty cycle. Methods for controlling the duty cycle of TOF MS have been developed. One or more ion gates with the function of transmitting or stopping the ion beam are placed in MS (Brenton et al., 2010). The mass range of ions injected into the TOF mass analyzer is controlled, increasing the repetition rate. But this only applies to a limited m/z range. The Encoded Frequent Pushing (EFP) method was developed to improve the duty cycle of high-resolution TOF MS (Willis et al., 2021). It was a spectral multiplexing technique. There were unique time intervals between each pulse to minimize the overlapping of ion peaks originating from different pushes. With the implementation of EFP, the duty cycle was increased in proportion to the increased pushing frequency. This meaned that the sampling efficiency of the continuous ion beam was higher. The signal intensity was enhanced by 10 times or more. The linear dynamic range was expanded by an order of magnitude. The LOD of octafluoronaphthalene (OFN) obtained is 100 fg. Trace analytes in matrices can be detected. The analysis data of the Egyptian mummy bandage extract also showed significant signal enhancement in EFP mode. Analytes that cannot be completely detected in no-EFP mode can be detected in EFP mode, such as chlorinated hydrocarbon analytes.

## FT-ICR Mass Analyzer

FT-ICR analyzer uses a magnetic field to trap ions. The m/z of ions is measured according to the cyclotron frequency of the ions in a given magnetic field (Comisarow and Marshall, 1974). FT-ICR analyzer has high mass accuracy and resolution (Bogdanov and Smith, 2005; Nagornov et al., 2014). It has become an important tool for the analysis of complex mixtures and molecular structures of large biomolecules (Sampson et al., 2009; Zeng et al., 2019). However, the instrument requires a strong magnetic field and an extremely high vacuum. This increases the cost for the operation of FT-ICR and limits its applications.

The detection circuit of FT-ICR includes the detection plates of the ICR cell, the amplifier and the analog-to-digital converter. Developments in the three components can improve the sensitivity of FT-ICR. The development of a cryogenic detection system provides circulation cooling for the cryopreamplifier inside the FT-ICR (Choi et al., 2012). This reduces the temperature of the detection circuit and decreases thermal noise, leading to the increase of the sensitivity. The number of ions needed to create a detectable signal is directly related to the capacitance of the detection circuitry (Limbach et al., 1993).

Kaiser et al. (2011) improved the electrical vacuum feedthrough and cable of the ICR cell in their custom-built 9.4 T FT-ICR mass spectrometer. The capacitance of the detection circuit was reduced and the sensitivity was increased. The instrument is very suitable for the analysis of complex mixtures, as well as proteomic analysis of large intact proteins. Identical signal-to-noise ratio (S/N) can be obtained for petroleum samples with 1/8 of the concentration and 1/2 of the ion external accumulation period compared to the former instrument. In the future, the detection circuit capacitance might be further reduced to improve the sensitivity, and novel ICR cell configurations can be explored.

Kaiser et al. (2014) applied an auxiliary RF waveform with the same amplitude and phase to all the rods of an ion accumulation multipole in the 9.4 T FT-ICR simultaneously. An m/z-dependent axial pseudo potential was created. An RF voltage ramp was constructed to control the ejection of ions from the accumulating octopole. Ions with different m/z reached the ICR cell at the same time. The abundances of the ions with high and low m/z were increased. The total number of fragment ions identified in proteomic analysis was increased by approximately 20% using the auxiliary RF ejection. For complex mixture analysis, the number of assigned elemental compositions was doubled. A transportable FT-ICR mass spectrometer was associated with a glow discharge ionization source to detect trace compounds in the air (Le Vot et al., 2016). Polycyclic aromatic hydrocarbons, such as naphthalene, acenaphthene and acenaphthene, were detected. The limits of detection are in the high ppb level.

## Tandem Mass Analyzers

Tandem Mass Analyzers refer to the combination of two or more mass analyzers. The characteristics of different analyzers are combined to make up for each other’s drawbacks (De Hoffmann, 1996; Sleno and Volmer, 2004). The separation, fragmentation and mass analysis of ions are realized in tandem mass analyzers. More structural information is obtained. The interference produced by the substrate molecules can be avoided. The background noise is greatly reduced. Tandem MS is particularly suitable for the analysis of samples with complex component systems and serious interference. It plays a vital role in biological and chemical analysis (Petrie et al., 2019; Rajski et al., 2019; Heiles, 2021).

### Combination of Quadrupole and Other Analyzers

#### QqQ

QqQ mass spectrometers consist of three quadrupoles and are one of the most common types of tandem mass spectrometers (Yost and Enke, 1979). The first quadrupole (Q1) and the third quadrupole (Q3) are used as mass filters. The second quadrupole (Q2) is used as a collision cell. QqQ MS has great advantages in quantification. It has become an indispensable analysis method for trace substances in complex matrices, such as the determination of pesticide residues and illegal additives in food (Zainudin et al., 2022), the quantification of drug metabolites (Li et al., 2022), the determination of pollutants in the environment (Zhu et al., 2020) and clinical research (Kramer et al., 2018). It will be more widely used in the detection of other trace substances in the future.

The use of selected ion monitoring (SIM) can get higher sensitivity (Jemal et al., 2003; Kato et al., 2012). For known compounds, only specific ions are scanned. The interference of other ions is eliminated. The signal intensity of specific ions is improved. High liquid chromatography (LC)-QqQ MS was used to quantify oak ellagitannins in Cognac in SIM mode (Gadrat et al., 2021). The LOD and LOQ at RSD <10% were 41.4 μg/L and 138 μg/L. This method has been successfully applied to the detection and quantification of 8 main oak ellagitannins in oak wood samples. A selected reaction monitoring (SRM) method was developed by Tsugawa et al. (2014) in a gas chromatography (GC)-QqQ MS to analyze the trimethylsilyl derivatives of 110 metabolites. Compared with single-quadrupole MS (Wei et al., 2010), SRM mode also provides high sensitivity and a wide dynamic range. Multiple reaction monitoring mass spectrometry (MRM-MS) is a technique for high-sensitivity target analysis. MRM is currently the most commonly used method for quantitative analysis by tandem MS. It has been widely used in the qualitative and quantitative analysis of small molecules (such as drugs and metabolites) (Lamont et al., 2018) and large molecules (such as proteins) (Lepretre et al., 2020). Q1 selects the targeted precursor ions of peptide according to m/z. In Q2, the precursor ions are fragmented by collision-induced dissociation (CID). Q3 detects selectively fragment ions at the specified m/z (Sherwood et al., 2009). Due to the high selectivity and duty cycle, MRM-MS has considerable sensitivity and multiplexing capabilities. Therefore, it can be used to detect and quantify specific molecules in complex mixtures. In proteomics, MRM-MS can be used to monitor and quantify peptides based on the expected fragment peaks generated by selected precursor ions of peptide.

Liu Z. et al. (2019) used MRM mode in a GC-QqQ MS to realize the quantitative analysis of 9 commonly encountered sulfonate esters in drug substances. The LOQ was between 0.10 and 1.05 ng/ml, which was much lower than the LOQs of other methods (2.5–1,500 ng/ml). The QqQ MS was used to analyze the anthocyanin derivatives in red wine under MRM mode (Zhang et al., 2020). The LODs and LOQs were 0.221–0.604 μg/L and 0.274–1.157 μg/L. Nijat, et al. (2020) used MRM method in high performance LC-tri-quadrupole LIT MS for quantitative analysis for 10 representative compounds as quality markers of Meiguihua Oral Liquid (MOS), including gallic acid, quercetin-3-Osophoroside, ellagic acid, sophoraflavonoloside, hyperoside, isoquercitrin, avicularin, astragalin, quercitrin and juglanin. The LOD was between 0.110 and 6.539 ng/ml and LOQ was between 0.443 and 32.693 ng/mL. A derivatization-assistedpseudo-multiple reaction monitoring with high CID voltage (HV-*p*-MRM) strategy was proposed by An et al. (2020), which can be used for rapid quantitative analysis of brassinosteroids (BRs) in different organs of rape flowers. A high CID voltage was applied in Q2. The precursor ions of the analyte were not easily fragmented under high CID voltage, while co-existing ions (impurity) of easy fragmentation can be fragmented. Finally, Q3 selected the precursor ions. Most of the co-existing ions were filtered out. The background signal was reduced, increasing the S/N of the analyte. The LODs of the mixed standards prepared in the ACN were between 1.49 and 6.56 pg/ml, and the LOQ were between 4.96 and 21.89 pg/ml. But the application of this method has limitations. It is suitable for the analysis of analytes with high stability.

#### Combination of Quadrupole and Ion Trap (Q-Trap)

Modifications on QqQ can make it directly into a combination of Q-trap. An auxiliary RF voltage is applied to the third quadrupole so that it can be used as a LIT. By making use of a novel curved linear collision cell, the speed of ion transmission and the ion capacity is increased. Cross contamination is effectively prevented. The sensitivity and resolution of the LIT can further be improved by adjusting the axial DC field and the radial RF field in the extraction region. Improvements in the ion transmission system and detector lead to better quantitative ability. The Q-trap instruments have been used in many fields, such as clinical trials (Roosendaal et al., 2019), pharmacokinetic studies (Ren et al., 2018) and targeted lipidome analysis (Khan et al., 2020).

In normal QqQ mode, the Q3 scan is used as a survey scan and a product ion scan is used as a dependent scan. In the LIT mode, an enhanced MS scan is used as a survey scan and an enhanced production scan is used as the dependent scan. Compared with the normal QqQ mode, the ion trap mode was used to analyze the extracted product ions in human urine, and the S/N was increased by about 60 times using a trapping time of 200 ms (Hopfgartner et al., 2003). It could be used to identify metabolites in complex matrices. When the LIT MS was scanning, the entrance RF-only section of the mass spectrometer was used as the accumulating ion trap, which could increase the duty cycle and increase the sensitivity by 20 times (Hager and Blanc, 2010). Sun et al. (2015) used the enhanced product ion mode of the LIT on the hydrophilic interaction liquid chromatography (HILIC) Q-Trap MS to identify and quantify four anthocyanins in red grape wine. The LOQ range was 0.05–1.0 ng/ml. An MS^3^ detection method was developed (Yin et al., 2021). The product ions generated in Q2 are captured by LIT. The selected product ions are further fragmented in the LIT and then detected. The LC-MS^3^ method is used for the quantification of methotrexate in human plasma. There is a good linear relationship in the range of 10–3,000 ng/ml (*R* ^2^ ≥ 0.995). Compared with the quantitative results of identical human plasma samples obtained by the LC-MRM method, the MS^3^ scan has higher sensitivity. The S/N for MS^3^ detection of methotrexate at 10 ng/ml is about 3 times that of MRM detection.

He, et al. (2017) placed the quadrupole in front of the dual-polarity LIT for ion transmission or mass selective transfer to the LIT. The LIT was used to accumulate ions. The types and amounts of substances involved in the reaction were precisely controlled. The occurrence of side reactions was reduced. The detection effect of reaction products was improved. Fang et al. developed a home-made Q-LIT tandem mass spectrometer for clinical biomarker analysis (Fang et al., 2021). The ions of interest were selected by the quadrupole into LIT. The interference of matrix ions was removed. The total number of ions entering the LIT was greatly reduced. Thus, the space charge effect was significantly reduced. The sensitivity was improved. This method can be used to analyze complex biological samples, which can significantly improve the efficiency and accuracy of analysis. It was used to measure small molecule disease markers in complex clinical samples.

#### 6.1.3 Combination of Quadrupole and TOF (Q-TOF)

In this combination, the Q3 of QqQ was replaced by TOF. It has the speed and sensitivity of a TOF and quantification capabilities similar to a QqQ (Andrews et al., 2011). It contains several different modes of quantification (Morin et al., 2013). In the SRMHR mode, the precursor ions are filtered by the quadrupole and fragmented in the collision cell. The TOF mass analyzer scans all product ions at high resolution (30 K). However, the sensitivity of the mass spectrometer is affected in order to achieve high resolution. The principle of SRMHS mode is the same as that of SRMHR. But the TOF scan resolution is limited to 15 K. The SRMHS enhanced mode used the same resolution (15 K) as SRMHS for SRM experiments. The difference is that the only product ion was specifically monitored and enhanced, which can significantly increase the sensitivity of the hybrid quadrupole and TOF mass spectrometer. The determination of biological analysis requires very sensitive mass spectrometers. Compared with TOF mode, the use of the SRMHS enhanced mode for sample quantification is more suitable for regulated bioanalysis.

Hou et al. (2020) used high performance liquid chromatography (HPLC) Q-TOF mass spectrometer to screen veterinary drugs and pesticides in eggs. Sulfachloropyridazine, sulfamethoxine, and fipronil sulfone were detected. When the residual concentration is greater than 10 μg/kg, Q-TOF and QqQ have comparable detection effects. When the residual concentration is less than 1 μg/kg, the detection effect of Q-TOF is better. A MRM method for determining peanut (Arachis hypogea) allergens in serum using Q-TOF was established (Hands et al., 2020). Compared with the QqQ, an Ara h 2 peptide was only detected by Q-TOF, and other peptide targets have similar assay sensitivities on the both mass spectrometers. Peptides can be repeatedly detected using the Q-TOF MRM method. The assay sensitivities were 0.53–1.3 fmol on-column.

Pesticides are easily degraded in the environment to produce products that are different from the original compound structure (Fenner et al., 2013). The high-resolution TOF mass analyzer is quite helpful for pesticide analysis (Bauer et al., 2018; Lee et al., 2020). Moreover, the Q-TOF can effectively improve the analysis accuracy of pesticides in the environment and reduce false positives in the analysis. Q-TOF can measure precise molecular weight and identify degradation products (Yuan et al., 2021). Q-TOF can be used with matrix-assisted laser desorption ionization (MALDI) (Minakata et al., 2017) and electrospray ionization (ESI) (Ling et al., 2020). Both ionization methods are suitable for biomolecules such as proteins. Q-TOF is often used in the identification and analysis of amino acid sequences in proteomics (Aili et al., 2016).

### Combination of Ion Trap and Other Analyzers

#### Ion Trap Arrays

A two-chamber ion trap with differential pressure regulation constructs a dual-pressure LIT (Second et al., 2009). Compared with the single trap, the first ion trap has a higher pressure. The trapping efficiency and the fragmentation efficiency of ions are improved. The second ion trap has a lower pressure. The scan rate and resolution are increased. Hybrid mass spectrometers with ion traps are widely used for the identification of peptides and proteins (Murugaiyan et al., 2017). The appearance of the dual-pressure LIT mass spectrometer has greatly increased the number of peptide and protein identifications. Ions can be more transported into the trap within a limited maximum injection time. The dual-pressure ion trap can obtain highly sensitive MS^2^ mass spectra. The probability of identification of low-abundance precursor ions is increased.

A differentially pumped dual linear quadrupole ion trap (DLQIT) mass spectrometer was developed (Owen et al., 2013). Tandem MS experiments were performed in the first trap, and then the generated product ions could be transferred to the second trap by axial ejection. The triggering of the two traps was integrated to ensure their synchronization. The most effective duty-cycle was obtained. It can promote the research of ionic structure through ionic molecular reaction and collision-activated dissociation (CAD), without mutual interference between experiments. A new type of RIT mass spectrometer with dual pressure chambers was designed by Huo et al. (2016). The skimmer, quadrupole, and inter-lens plate can act as a quadrupole linear ion trap (Q-LIT) in the first vacuum chamber, and RIT was in the second vacuum chamber. In the Storage Quadrupole Linear Ion Trap-Rectilinear Ion Trap (SQLIT-RIT) mode, Q-LIT was used for ion storage and isolation under high pressure, while RIT was used for analysis. In traditional RIT MS with dual pressure chambers, the linear quadrupole and octupole were operated only as ion guides (Fico et al., 2007; Wang et al., 2012). Compared to with the traditional Q-RIT mode, the signal strength of the SQLIT-RIT mode was increased by a factor of 30, and the LOQ of imatinib was reduced by an order of magnitude to 50 ng/ml.

Selective ion transfer and accumulation techniques can be performed in ion trap arrays, which can effectively improve the detection sensitivity of the low-abundance ions. A dual ion trap mass analyzer that can detect ions with a high m/z (m/z > 6,000) was developed (Hsu et al., 2013). Step scanning of the trapping frequency for a sample was performed in a QIT. The selected ions ejected from the QIT were trapped by the LIT. Compared with the dual QIT, this device exhibited higher trapping efficiency. For large biomolecular ions with high m/z, ions could be transferred from the QIT to the LIT at an efficiency of about 60%, which was the highest transfer efficiency in dual ion trap device for high-mass ions at that time. It may be a convenient instrument for studying top-down proteomics to detect intact proteins. Wang et al. used the first ion trap to store the injected ions (Wang et al., 2014). The targeted ions were then selectively ejected into the second ion trap. After several mass selective transmissions, a certain amount of the targeted ions were accumulated in the second ion trap. They were subsequently detected. The sensitivity of was enhanced by the accumulation of low-abundance ions in the ion trap. This method may be further applied to the study of gas phase ion reactions. Product ions can be transferred and accumulated in the second ion trap to prevent further reactions or fragmentations. Kamsap et al. (2015) constructed a double linear trap. The ion cloud was unidirectionally transferred from the fist ion trap to the second ion trap and accumulated. The number of ions in the second ion trap was increased by the accumulation of multiple cycles, thereby improving the S/N.

**FIGURE 3:**
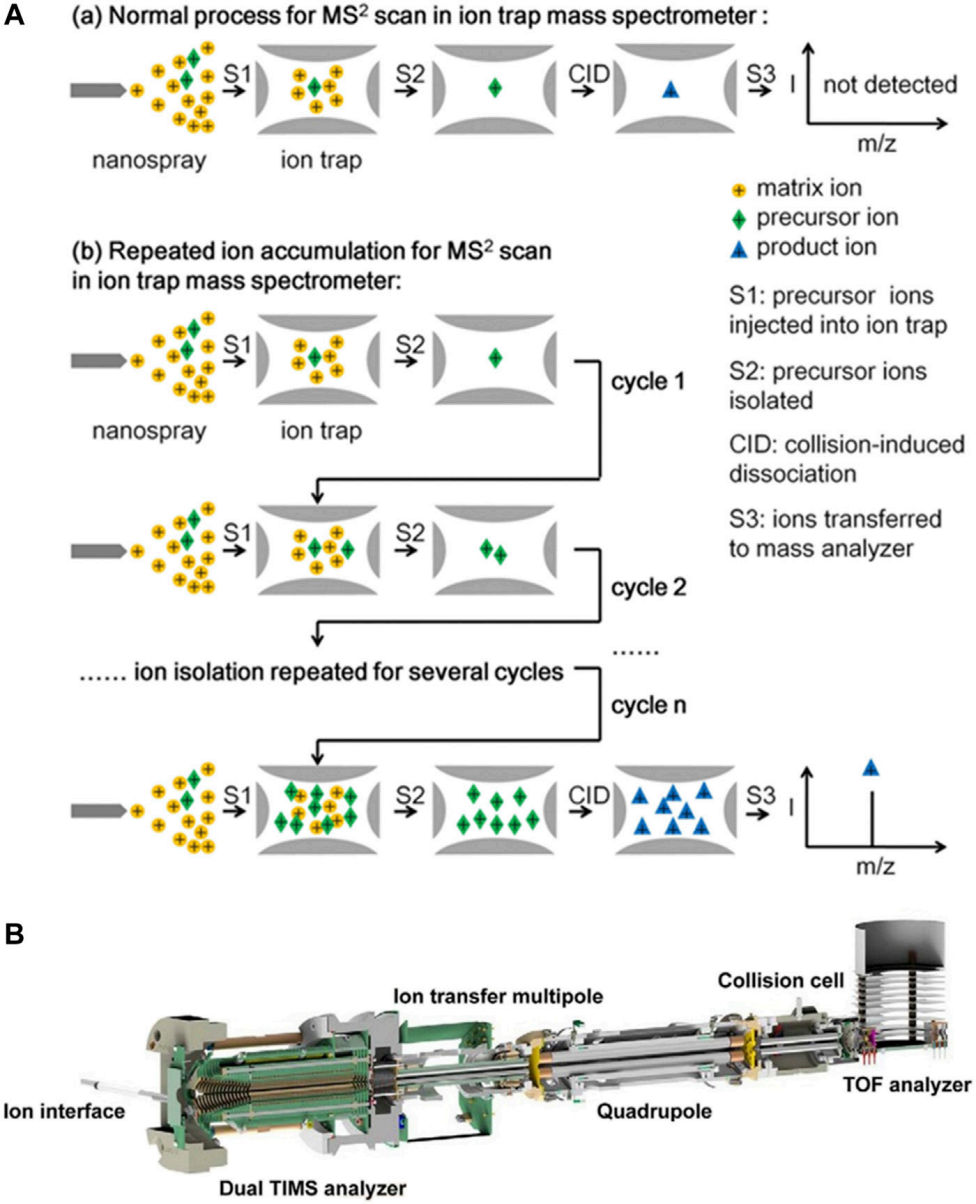
**(A)** Normal procedure for MS^2^ scan in ion trap mass spectrometer (a) and procedure of repeated ion accumulation by ion trap mass spectrometer (b). Adapted with permission from Si, X. Y., Xiong, X. C., Zhang, S. C., Fang, X. and Zhang, X. R. (2017). Anal. Chem. 89, 2,275–2,281 (Si, et al., 2017). Copyright 2017 American Chemical Society; **(B)** The dual TIMS and Q-TOF tandem mass spectrometer. Adapted with permission from Meier, F., Brunner, A.-D., Koch, S., Koch, H., Lubeck, M., Krause, M., et al. (2018). Mol. Cell. Proteomics 17, 2,534–2,545 (Meier et al., 2018). Copyright 2018 Elsevier.

A technique for repeated ion accumulation in the ion trap MS was developed to improve sensitivity (Si et al., 2017). The injected precursor ions were isolated and stored in the high-pressure ion trap before CID (Figure 3A). Repeating the ion injection and isolation steps, a large number of precursor ions were accumulated in the LIT. When ions were repeatedly accumulated for 25 cycles, the detection sensitivity of various molecules was increased 3–22 times. Low-abundance ions could be stably detected at the single-cell level. This technique is more suitable for targeted analysis of low-concentration analytes. It may further identify and quantify more metabolites, peptides or proteins in single-cell analysis. He et al. (2019) developed a mass spectrometer with three linear ion traps connected by a quadrupole deflector. Cations and anions could be generated and introduced from either side of the mass spectrometer to the LIT. Both reaction intermediate ions and productions were selectively transferred and accumulated in the last ion trap. After multiple rounds of reactions and accumulations, the signal intensity was significantly enhanced. The intermediate ions and product ions could be analyzed by tandem MS, which could quickly characterize the reaction with high confidence. This method can be used in biochemistry, such as rapid screening of drug-protein interactions.

Miniaturized mass spectrometers have achieved rapid development in the past few years. But the sensitivity was reduced due to size reduction. Strategies for ion trap arrays have been developed to increase the storage capacity of ion traps to increase sensitivity. In addition, miniaturized CIT array was constructed to expand the total space for ion capture (Tabert et al., 2003). The CIT array with multiple parallel channels has the potential for high-throughput analysis of multiple samples at the same time. Ding’s group developed an ion trap array built using the printed circuit board (PCB) fabrication technology (Li et al., 2009). The analysis speed and sensitivity of MS have been doubled improved. High-throughput MS analysis was achieved. Xu et al. (2014) explored an ion trap array called an “ion sponge”. The 3D array with hundreds of ion traps was constructed from meshes with narrow wires. Ion transfer and analysis were performed between the layers. Yu et al. (2016) proposed a novel toroidal-cylindrical ion trap mass analyzer, which consists of an outer toroidal ion trap (T-trap) and an inner CIT. The T-trap is mainly used for ion capture and storage during the MS/MS period. The precursor ions are selectively ejected from the T-trap into the CIT and then trapped and fragmented in the CIT. A more efficient MS/MS procedure can be achieved. A miniature mass spectrometer with dual linear ion traps was established to develop a comprehensive scanning mode for tandem MS (Liu X. et al., 2019). It has great potential for comprehensive qualitative and quantitative analysis.

Silveira et al. (2017) developed a dual TIMS to separate the accumulation process of ions from the mobility analysis process. The elongated TIMS tunnel was composed of a trap, a transfer region and a TIMS analyzer. Ions are trapped and accumulated in the trap. Then, the ions are transferred into the TIMS analyzer for mobility analysis. At the same time, the trap is filled with the next batch of ions. Through parallel accumulation and analysis of ions, the duty cycle is close to 100%. The ion utilization efficiency and sensitivity are improved. Parallel accumulation-serial fragmentation (PASEF) technique is used in a dual TIMS and Q-TOF tandem mass spectrometer (Figure 3B), improving the sequencing speed without loss of sensitivity (Meier, et al., 2018). First, the accumulation and mobility analysis of ions are parallel to avoid ion loss. Then, the isolation of the ions by the quadrupole and the elution of the ions after the mobility separation are simultaneous. It is used for the analysis of lipids, proteins and complete macromolecular assemblies. Liu et al. (2018) further combined two TIMS analyzers and developed a tandem TIMS (TIMS-TIMS) analyzer. TIMS-TIMS is incorporated in a hybrid quadrupole time-of-flight (QqTOF) mass spectrometer for rapid and sensitive biological analysis (Liu et al., 2020).

#### Combination of Ion Trap and FT-ICR

In this combination, ions are pre-accumulated in the ion trap before being injected into the FT-ICR mass analyzer. Compared to directly trapping ions in the ICR cell, the pre-accumulation and storage of ions improves the sensitivity, scan rate and duty cycle (Senko et al., 1997; Syka et al., 2004). Weisbrod et al. (2013) replaced the single-cell LIT with a dual-cell LIT. The dual-cell LIT delivers more ions to the ICR cell through multiple filling, which provides high ion capacity and ejection efficiency (Hendrickson et al., 2015). Weisbrod et al. (2017) equipped a multipole storage device (MSD) in a hybrid system of dual-cell LIT and customized FT-ICR. The ion-ion reaction was repeated with a relatively small number of precursor ions. More product ions were accumulated before being transferred to the ICR cell for mass analysis, which improved the S/N. The reaction products could be accumulated in the MSD until the maximum sequence coverage was reached.

**FIGURE 4:**
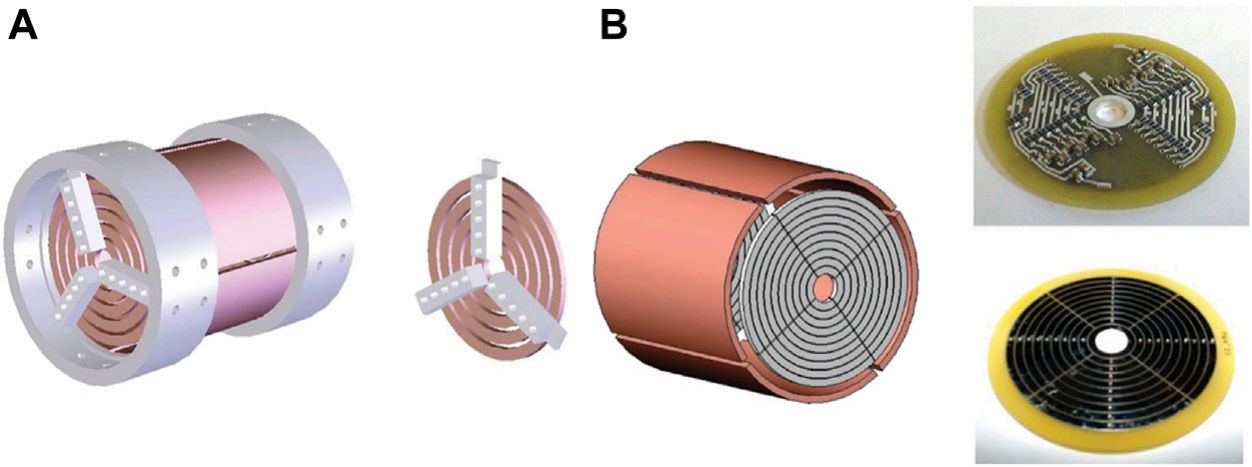
Novel designs of the closed-cylindrical FT-ICR cell. **(A)** Design of the TREC. Adapted with permission from Weisbrod, C. R., Kaiser, N. K., Skulason, G. E. and Bruce, J. E. (2008). Anal. Chem. 80, 6,545–6,553 (Weisbrod, et al., 2008). Copyright 2008 American Chemical Society; **(B)** Design of the eTREC. Adapted with permission from Weisbrod, C. R., Kaiser, N. K., Skulason, G. E. and Bruce, J. E. (2010). Anal. Chem. 82, 6,281–6,286 (Weisbrod, et al., 2010). Copyright 2010 American Chemical Society.

Ostrander et al. (2000) added a central trapping electrode on the accumulation cell between the shutter and skimmer in the 3.0 T ESI/FT-ICR mass spectrometer. A negative potential was applied to the central trapping electrode. The well depth was increased. The ions were accumulated. The S/N was increased by 30 times. Weisbrod et al. (2008) developed a novel type of FT-ICR cell, namely the trapping ring electrode cell (TREC). The trap plates were divided into five concentric ring electrodes (Figure 4A). The DC voltages of the ring electrodes were controlled independently, so that the ions were excited to a large cyclotron radius. Compared to the way in which the same voltage applied to all ring electrodes during detection, the signal intensity was enhanced by 10 times with the TREC. The ability for protein identification was improved (Kaiser et al., 2009). Weisbrod et al. (2010) developed an excite-coupled TREC (eTREC) (Figure 4B). The RF excitation was coupled to the trapping rings of the TREC. The *z*-axis ejection was effectively reduced. The entire ion population had a more uniform post-excitation radius. The sensitivity was improved by more than 50%. The LOD was greatly reduced.

### Combination of Orbitrap and Other Analyzers

**FIGURE 5:**
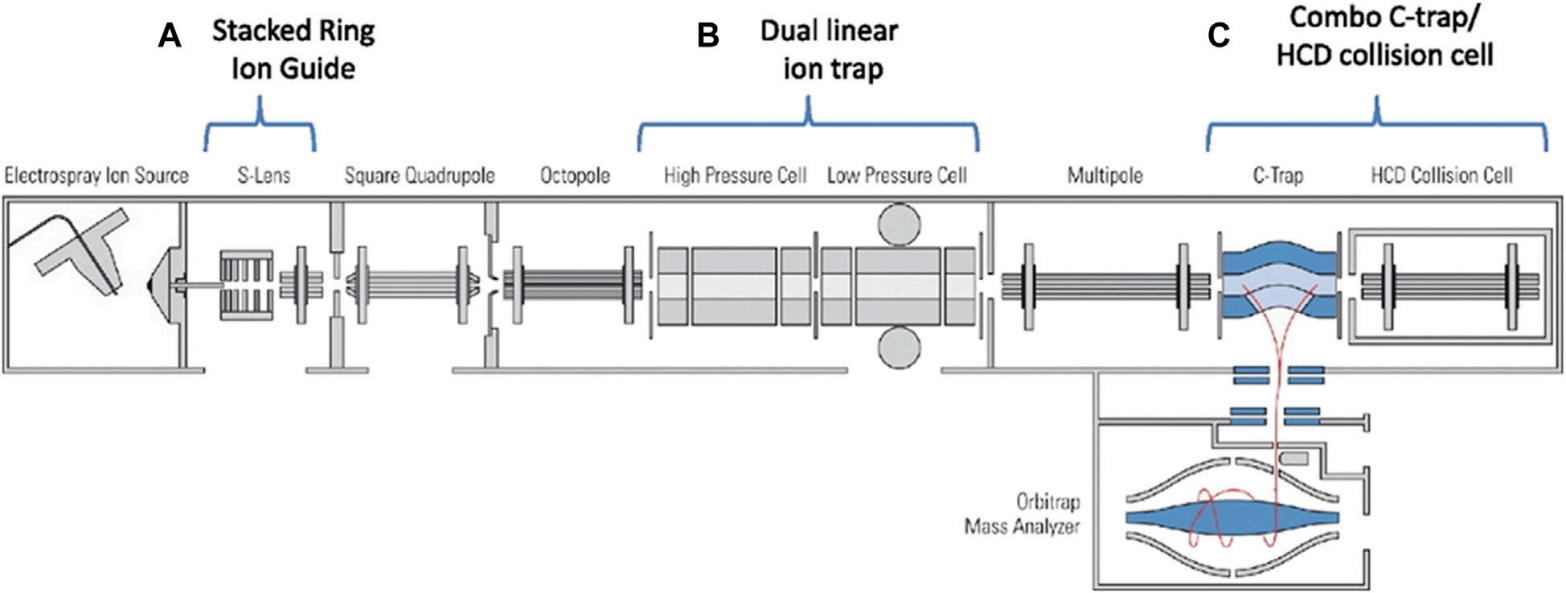
Schematic of the hybrid dual LIT and Orbitrap mass spectrometer. Adapted with permission from Olsen, J. V., Schwartz, J. C., Griep-Raming, J., Nielsen, M. L., Damoc, E., Denisov, E., et al. (2009). Mol. Cell. Proteomics 8, 2,759–2,769 (Olsen, et al., 2009). Copyright 2009 Elsevier.

Orbitrap is a mass analyzer with ultra-high mass resolution, usually combined with other mass analyzers. The C-trap can be used as a collision cell to obtain fragment ions similar to the QqQ (Olsen et al., 2007). This fragmentation technique is higher energy collisional dissociation (HCD). The HCD requires a higher RF voltage, which leads to a reduction in trapping efficiency in the low-mass range. In the hybrid LIT-Orbitrap mass spectrometer, an octopole collision cell is installed at the other end of the C-trap to trap fragment ions in a very wide mass range. The dual trap is introduced (McAlister et al., 2010; Olsen et al., 2009). The hybrid dual LIT and Orbitrap mass spectrometer is shown in Figure 5. The characteristics of the dual trap can be combined to improve the sensitivity. The detection sensitivity and mass resolution of large protein assembly ions were improved by using high HCD voltage offsets, higher gas pressures and xenon instead of nitrogen in the HCD cell (Rose et al., 2012). The ions are trapped in the HCD cell before returning to the C-trap, which can more efficient desolvation and capture large proteins and significantly improve sensitivity.

LIT and Orbitrap tandem mass spectrometer can be used for multi-residue screening of complex samples and compound confirmation (Kosma et al., 2021). It is also an indispensable tool in fields such as proteomics (Stryinski et al., 2019) and metabolomics (Qin et al., 2020). LIT-Orbitrap has the double confirmation function of MS^n^ and precise molecular weight (Kite, 2020). The precise molecular weight and MS^2^ spectrum of many peptides after protein digestion can be determined. After searching the database, the protein is identified. The false positive rate is greatly reduced. The added ETD function is mainly used for the analysis of phosphorylation sites, which makes up for the deficiency of CID in the post-translational modification function (Riley et al., 2016). High-resolution mass spectrometry (HRMS) can distinguish background matrix interferences, which can be used for quantitative and high-reliability screening of complex matrix samples.

Coupling a quadrupole in front of Orbitrap can increase the selectivity of Orbitrap (Nolting et al., 2019). Another important improvement is that the filling of the C-trap and the acquisition of Orbitrap can be run in parallel. The duty cycle of the analysis is significantly increased, usually reaching a level above 90%. The hybrid mass spectrometers, such as the Q-Orbitrap mass spectrometer and the hybrid Q-Orbitrap and ion trap (Q-Orbitrap-IT) mass spectrometer, uses high-capacity multipole ion traps to accumulate ions before analysis (Senko et al., 2013). The Q-Orbitrap-IT mass spectrometer is used to perform accumulated ion monitoring (AIM) for targeted proteomics (Cifani and Kentsis, 2017). The precursor ion filtering of the quadrupole, the quantification of the precursor in the orbitrap, and the parallel identification of fragmentation spectra in the LIT are combined. Researchers had developed SIM stitching techniques based on the LIT or QMS on high-resolution ion trap mass spectrometers such as ICR and Orbitrap (Southam et al., 2007; Kang et al., 2019). Multiple narrow SIM segments were collected together after acquisition to produce a wide range of spectra. Each SIM window was composed of relatively few ions to minimize space charge effects. Compared with the unsegmented broad full peak, this method improved the S/N in a wider m/z range. This method is widely used for MS measurement of any complex mixture. Especially for biological samples, it is beneficial for both metabolomics and proteomics.

In recent years, HRMS has been rapidly developed. HRMS is widely used for the analysis of residues and contaminants in food and environmental samples. A Q-Orbitrap system has been used for the screening of pesticide residues in fruits and vegetables (Ucles et al., 2017). The LOQ reached 10 μg/kg or lower. GC-Q-Orbitrap can monitor organic pollutants in wastewaters (Dominguez et al., 2020). This method can be used for the determination of 15 polycyclic aromatic hydrocarbons in wastewaters. LOQ at ppt (0.03–0.70 ng/L) level can be obtained. 100 pesticides and pollutants in different complex food matrices were screened and quantified by GC-Q-Orbitrap MS with full scan mode and GC-QqQ method with MRM mode (Belarbi et al., 2021). Among them, 86 pesticides and pollutants have lower LODs obtained by GC-Q-Orbitrap MS. The GC-Q-Orbitrap method has the highest quantitative sensitivity for most pesticides in wheat. The LOQs were between 0.1 and 4 μg/kg.

HRMS is also widely used in the field of metabolomics. A Q-Qrbitrap MS is used to determine real plasma extracts (Grund et al., 2016). The quantitative results of protease inhibitors, tyrosine kinase inhibitors, metanephrines and steroids show that HRMS is a reliable and sensitive quantitative instrument. The quantitative performance of HRMS is comparable to QqQ MS. HR-Q-Orbitrap MS and parallel reaction monitoring (PRM) used for the quantitative analysis of immunoglobulin G (IgG1, G2, G3 and G4) in a small amount of human blood plasma (Huang and Pan, 2017). The LODs were 2.5 μg/L for IgG1, 3.4 μg/L for IgG2, 1.2 μg/L for IgG3 and 4.0 μg/L for IgG4. For generic surrogate peptide DTL, the LOD of all IgGs was 0.4 μg/L. Compared with Q-TOF MS in MRM mode, it has higher sensitivity. HR-Q-Orbitrap-MS in PRM mode has higher sensitivity. Synthetic cathinones in urine and oral fluid were determined by an Orbitrap and a QqQ MS (Pascual-Caro et al., 2020). The LODs obtained by the Orbitrap were between 0.005 and 0.035 ng/ml. The LOQs of most analytes obtained by the Orbitrap were 0.050 ng/ml, and the LOQs of ethcathinone, buphedrone, and 4-methylcathinone (4-MEC) were 0.200 ng/ml. The LODs obtained by the QqQ were between 0.040 and 0.160 ng/ml. The LOQs obtained by the QqQ were between 0.020 and 0.070 ng/ml. The QqQ can get lower LOD and LOQ.

## Conclusion and Perspective

In the past 2 decades, mass analyzer techniques developed rapidly. Each kind of mass analyzers has its own advantages and limitations. The choice of mass analyzers depends on the application field and the corresponding detection requirements. This review discusses the significant development of different mass analyzers in the improvement of sensitivity, including quadrupole, ion trap, TOF and their combinations. The sensitivity of the quadrupole mass spectrometer is usually improved by increasing the transmission efficiency of the quadrupole. The transmission efficiency of quadrupole mass analyzer can be improved by the introduction of the delayed DC ramp and the modification of the first stability diagram. The developed techniques of ion trap mass analyzer are discussed such as the modification of the geometric shapes of the ion trap and the application of additional signals. The ejection efficiency and trapping efficiency of the ion trap can be improved. The targeted ions are effectively accumulated. But if a large number of ions are stored in the ion trap, there will be a strong Coulomb interaction between them, resulting in space charge effects. Thus, some methods have been developed to reduce the space charge effects. The sensitivity of TOF MS is usually improved by focusing the ion packets and improving the duty cycle.

Tandem mass spectrometer combines the advantages of different analyzers to improve the performances of mass spectrometers. Quadrupole is often placed in front of other mass analyzers to pre-select targeted ions. Interferences from matrix ions are removed, significantly reducing chemical noises. The sensitivity of the ion trap tandem MS is related to the number of effective ions trapped. Thus, the improvement of the trapping efficiency and the accumulation of the targeted ions are feasible methods to obtain better sensitivity. Ion traps are usually combined with other mass analyzers to effectively accumulate ions before analysis. External accumulation and storage of ions improved duty cycle and sensitivity. Some methods have been developed to reduce the effect of space charge effects. These advancements will continue to drive the development of current MS.

The performances of mass spectrometers with different mass analyzers are compared. In general, QqQ mass spectrometer has the highest sensitivity. The QqQ is the most commonly used mass analyzer for quantitative detection due to its higher accuracy. It is widely used in many fields such as food safety, clinical medicine and proteomics. However, it has insufficient qualitative capability, such as high mass resolution and MS^n^. Ion trap can realize MS^n^ analysis, which is beneficial to qualitative analysis. It has wider applications in basic scientific research. The Q-Trap system combines the scanning mode of the tandem quadrupole with the scanning mode of the LIT. It can provide highly sensitive quantitative analysis data and qualitative analysis data. High resolution MS has the functions of high mass resolution and precise molecular weight. It can confirm the structure of biological samples and realize the analysis of biological macromolecules such as proteins. The main types of high resolution MS are TOF, FT-ICR and Orbitrap. Compared with other high-resolution mass spectrometers, the resolution of TOF is lower, and the accuracy of its measurement data is greatly affected by operating conditions. The FT-ICR mass spectrometer has ultra-high resolution and high-mass accuracy. But its limitations, such as expensive price, huge volume, high operating cost and complicated operation, make it difficult to be widely used in various fields, especially as a conventional analytical instrument. The Orbitrap systems provide high mass resolution and mass accuracy in a wide mass range including the mass of small molecules. A wide range of compounds and small molecules can be detected during target analysis and non-target analysis without loss of selectivity or sensitivity. In recent years, the development of HRMS has made up the sensitivity gap with QqQ, and even provides better selectivity and sensitivity than detection of QqQ with SRM mode.

The selection of suitable mass analyzer is dependent on the applications interested in. When analyzing samples with complex matrices, a quadrupole would be useful to reduce the interferences from the matrices. For further quantitative analysis of trace components in complex samples, QqQ or Q-Orbitrap would be a good choice due to their high sensitivity and quantitative ability. In qualitative studies, ion trap is commonly used to conduct MS^n^ fragmentation of the targeted molecules so as to investigate their structures. Orbitrap is also used to obtain the accurate mass of the targeted molecules, which reveals the elemental compositions of the molecules.

Mass spectrometers will continue to pursue high sensitivity. Novel methods are needed to further improve the performance of MS for accurate qualitative and quantitative analysis of trace substances in complex matrices. At the same time, improved pre-processing technology and ionization technology can be combined. The rapid development of MS will promote its applications in different fields such as clinical testing, environmental monitoring, and life sciences. In addition, mass spectrometers are developing towards portable and miniaturized devices. Compared with the other mass analyzers, LIT is more easily miniaturized. In recent years, there has been a great demand for miniaturized mass spectrometers in food safety, environmental monitoring, military and other fields. In the future, miniaturized mass spectrometers would be more practical. The miniaturization might be achieved by optimization of the electrode structure and hardware circuit, without losing the performance of instruments, such as sensitivity and quantitative ability.
